# Mathematical modeling of palatal suture pattern formation: morphological differences between sagittal and palatal sutures

**DOI:** 10.1038/s41598-021-88255-y

**Published:** 2021-04-26

**Authors:** Nobuhide Shibusawa, Yoshie Endo, Naoki Morimoto, Ichiro Takahashi, Takashi Miura

**Affiliations:** 1 Faculty of Dental Science, Kyushu University, Fukuoka, Japan; 2 Academic Society of Mathematical Medicine, Faculty of Medicine, Kyushu University, Fukuoka, Japan; 3 Department of Anatomy and Cell Biology, Kyushu University Graduate School of Medical Sciences, Fukuoka, Japan; 4 Laboratory of Physical Anthropology, Department of Zoology, Graduate School of Science, Kyoto University, Kyoto, Japan; 5 Section of Orthodontics and Dentofacial Orthopedics, Faculty of Dental Science, Kyushu University, Fukuoka, Japan

**Keywords:** Anthropology, Anatomy, Musculoskeletal system, Oral anatomy

## Abstract

The median palatal suture serves as a growth center for the maxilla; inadequate growth at this site causes malocclusion and dental crowding. However, the pattern formation mechanism of palatal sutures is poorly understood compared with that of calvarial sutures such as the sagittal suture. In the present study, therefore, we compared the morphological characteristics of sagittal and palatal sutures in human bone specimens. We found that palatal suture width was narrower than sagittal suture width, and the interdigitation amplitude of the palatal suture was lower than that of the sagittal suture. These tendencies were also observed in the neonatal stage. However, such differences were not observed in other animals such as chimpanzees and mice. We also used a mathematical model to reproduce the differences between palatal and sagittal sutures. After an extensive parameter search, we found two conditions that could generate the difference in interdigitation amplitude and suture width: bone differentiation threshold $v_c$ and growth speed *c*. We discuss possible biological interpretations of the observed pattern difference and its cause.

## Introduction

**Figure 1:**
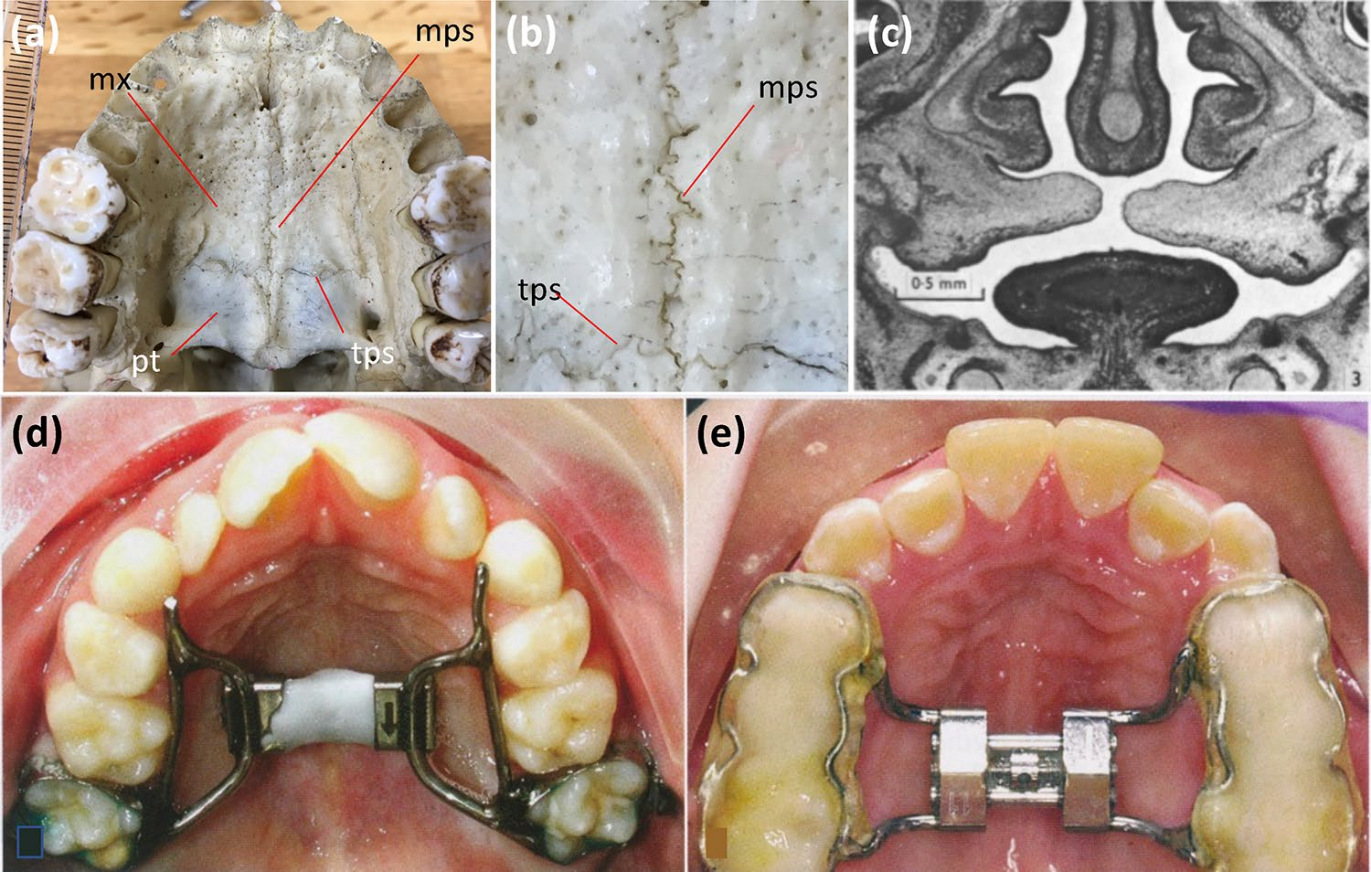
Anatomy of the palatal suture: association with development and clinical treatment. **(a)** Palatal suture. The median palatal suture is at the midline of the hard palate, while the transverse palatal suture is at the interface between the maxilla and palate. **(b)** High magnification view of **(a)**. **(c)** Developing palatal suture^12^. Ossification starts from both sides of the palate and proceeds to the middle. **(d)** Stenotic arch due to hypoplasia at the median palatal suture. **(e)** After treatment with a rapid expansion device (modified from^23^). *mx* maxilla, *mps* mid palatal suture, *pt* palatal bone, *tps* transverse palatal suture.

The palatal suture is the suture between the left and right maxilla and palatal bone^1^. It is composed of a median palatal suture, present at the midmaxillary region, and a transverse palatal suture that forms the border between the palatal bone and the maxillary bone (Fig. 1a,b). The majority of the hard palate consists of maxilla^2^; thus, the transverse palatal suture lies in the posterior third of the hard palate. The morphology and development of the palatal suture was originally described by direct observation of bone specimens^3,4^ and later by radiographic observations^5–7^. In forensic science, the palatal suture is an age indicator^8^, similar to the calvarial suture^9^. Both the calvarial^2^ and palatal sutures^10^ have been reported to have fractal characteristics.

**Figure 2:**
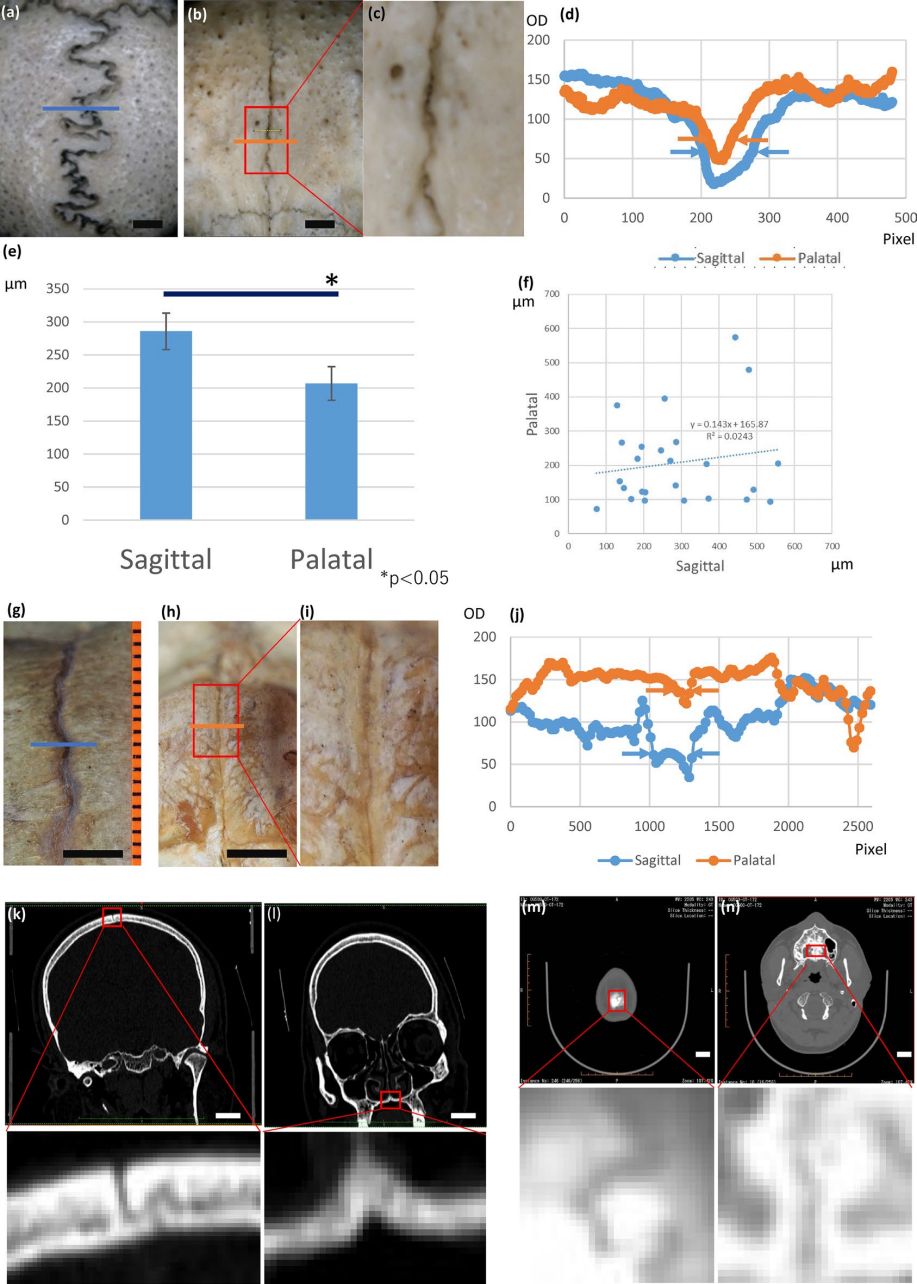
The difference in suture width between human sagittal and palate sutures. **(a)** Sagittal suture of the adult human skull; suture width is less than 1 mm. **(b)** Palatal suture of the same individual. The scale is the same as in **(a)**. The suture width is less than 500 $\upmu$m. **(c)** High-magnification view of **(b)**. A fine curvature was observed inside. **(d)** Profile plot of the suture line area. The midpalatal suture (orange) is narrower and shorter in wavelength than the sagittal suture (blue). **(e)** The widths of the sagittal and palatal sutures were significantly different. **(f)** Sagittal and palatal suture widths were not strongly correlated. **(g)** Sagittal suture of a newborn human skull. **(h)** Palatal suture of the same newborn human skull. **(i)** High-magnification view of **(h)**. **(j)** Optical density distribution of the suture area. The midpalatal suture (orange) is narrower than the sagittal suture (blue). **(k)** Sagittal suture in 3DCT of an adult human head (coronal section). **(l)** Palatal suture of the same individual (coronal section). **(m)** Sagittal suture in 3CDCT of a human head (horizontal section). **(n)** Palatal suture of the same individual (horizontal section). OD: optical density. Scale bars: **(a,b,g,h)** 5mm, **(k–m)** 2 cm.

The palatal suture plays an important role in the growth and development of the maxilla^11^. During palate development, palatal shelves first appear from both sides of the floor of the maxilla; these later become fused to form the soft and hard palate (Fig. 1c,^12^). Subsequently, the palatal suture closes in a similar manner to calvarial suture closure, although considerable individual variation exists within this process^5,13^. The maxilla develops via membranous ossification, similar to calvarial bone development, and many genes are involved in both calvarial and palatal suture development^14^ (e.g., *Fgfr2*^15,16^, *Nell1*^17^, *Tgf* $\beta$1^18^, *Basonuclin-2*^19^). In addition, physical factors (e.g., laser irradiation^20^ and mechanical force^21,22^) are involved in the ossification process. The driving force behind maxillary growth at the palatal suture is believed to be the growth of the tongue and nasal cavity, whereas the driving force behind bone growth at the calvarial suture is the growth of the brain^11^.

Palatal suture research is clinically important from an orthodontics perspective^23^. For example, maxillary growth failure can cause malocclusion and dental crowding, which are relatively frequent anomalies. Rapid maxillary expansion (gradual expansion of the midpalatal suture using a mechanical device during the juvenile stage) is frequently used to treat maxillary growth failure (Fig. 1d,e). Palatal suture development is also related to the treatment of cleft palates^24,25^. To optimize such treatments, understanding palatal suture development is clinically important^6,26–29^. However, to date, palatal suture research is less well advanced than calvarial suture research.

In the present study, we examined the morphology of palatal sutures and compared them with the morphology of sagittal sutures, i.e., the calvarial suture that has been most extensively studied. Firstly, we quantified the pattern of palatal sutures using Fiji. In examinations of human bones, we found that the palatal suture was narrower than the sagittal suture, and that the interdigitation amplitude of the palatal suture was smaller than that of the sagittal suture. Secondly, using a mathematical model of suture pattern formation, we applied numerical simulations to understand this difference. Our model consisted of an interface equation and a diffusion equation, which contained two variables: the bone differentiation state *u* and the bone differentiation promoting factor *v*. As a result of parameter screening for a condition that reproduces the short wavelength of palatal suture curvature, we found that palatal suture patterns could be reproduced by the model when the critical values of FGF $v_c$ or domain growth speed *c* were changed. Based on these results, we consider the biological relevance of the conditions that reproduce the characteristics of palatal suture shape.

## Results

### The palatal suture is narrower than the sagittal suture in the human skull

To compare the morphological characteristics of sagittal and palatal sutures in human bone specimens, the specimens were first digitized using a binocular microscope and digital camera, and then the images were calibrated and suture widths were measured. The palate suture was narrower and had finer curvature than the sagittal suture (Fig. 2a–c). This tendency was confirmed by examining the brightness distribution of the images (Fig. 2d). In addition, the widths of the sagittal and palatal sutures in 26 human skull samples were manually measured. Although certain amount of variation exists among specimens, statistically significant difference was detected (Fig. 2e, Student’s t-test). Correlation analysis of the widths of sagittal and palatal sutures showed that they are relatively independent (Fig. 2f).

To investigate the developmental stage at which this difference emerged, we also observed a skull specimen of a juvenile human provided by Kyoto University ($n=1$). Although we observed a palatal–sagittal suture width difference in these specimens (Fig. 2g–j), a statistical difference was not detected due to the small number of samples; nevertheless, the width difference seemed to already exist at the newborn stage.

We also measured the suture morphology using radiological data since measurements of bone specimens can be influenced by soft tissue absence. We visualized sagittal and palatal sutures in a free 3DCT volume dataset ($n=15$)^30^. For the frontal section of the sagittal suture, we chose a section that included both the left and right mandibular condyles. For the frontal section of the palatal suture, we chose a section that included the zygomatic arch’s dorsal edge. For the horizontal sections, we chose the highest point, which included the sagittal suture, and a section that included the surface of the hard palate for observation of the palatal suture. From our analyses of these 3DCT data, we found that the sagittal suture was wider than the palatal suture, which confirmed our earlier observations (Fig. 2k–n).

### Widths of palatal and sagittal sutures are not different in other species

Next, we observe the species difference of this phenomenon.

**Figure 3:**
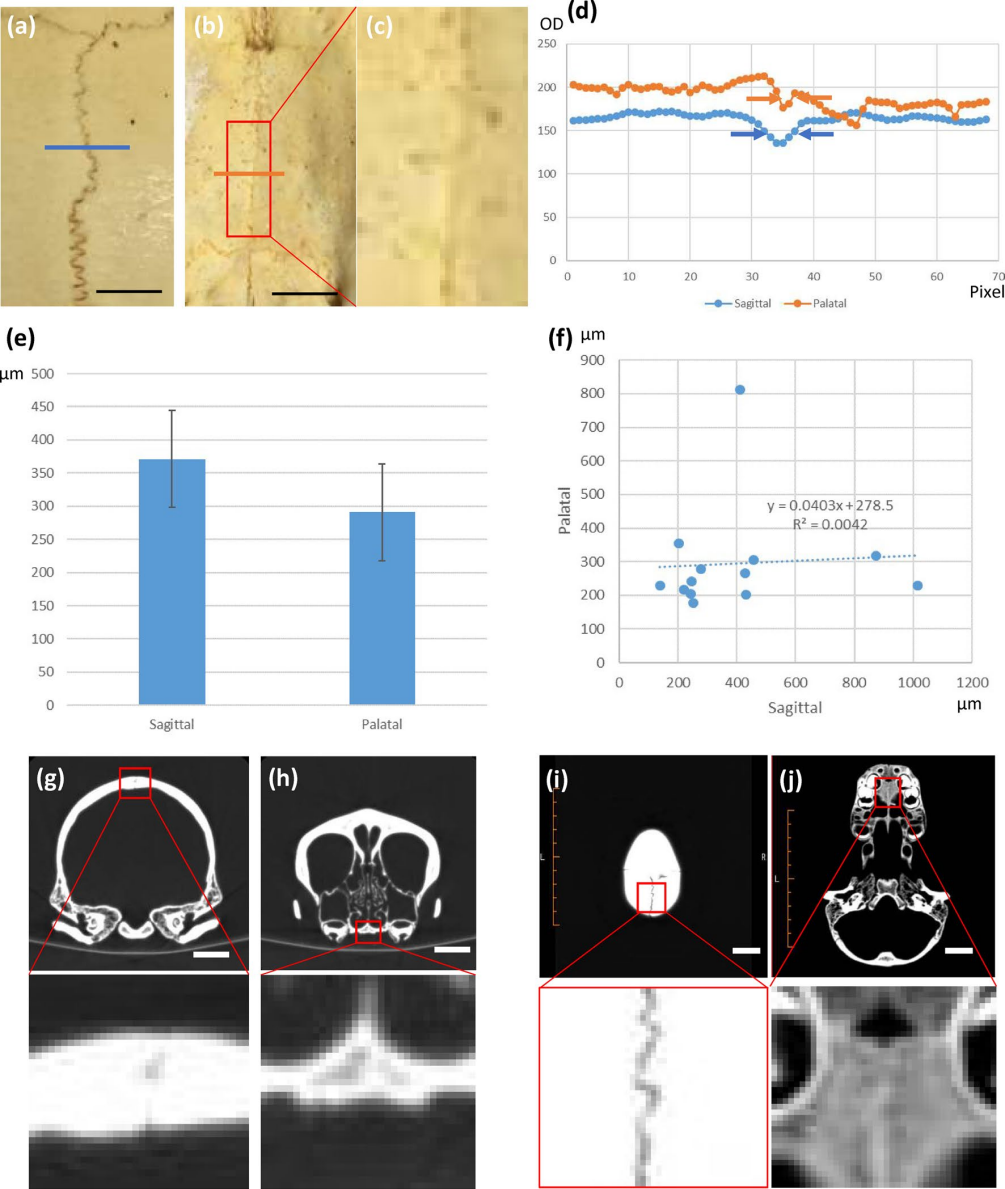
Sagittal and palatal suture widths are not significantly different in the chimpanzee skull. **(a)** Sagittal suture of a juvenile chimpanzee skull. **(b)** Palatal suture of the same sample. **(c)** High-magnification view of **(b)**. **(d)** Profile plot of the suture line area. The difference between the sagittal suture and palatal suture is not clear. **(e)** Manual measurement of suture widths. A statistically significant difference between the sagittal and palatal suture widths was not detected. **(f)** Correlation between sagittal and palatal suture width. **(g)** Sagittal suture of the chimpanzee skull: frontal section. **(h)** Palatal suture of the chimpanzee skull: frontal section. **(i)** Sagittal suture of the chimpanzee skull: horizontal section. **(j)** Palatal suture of the chimpanzee skull: horizontal section. *OD* optical density. Scale bars: **(a,b)** 500 ${\upmu }$m, **(g–j)** 2 cm.

We first compared the width of palatal and sagittal sutures in chimpanzees, i.e., the most closely related living primate to humans (Fig. 3). We used juvenile chimpanzee skulls ($n=13$) since skull sutures are generally closed in adult primates. Although we found that the sagittal suture tended to be wider than the palatal suture in these skulls, the difference was not statistically significant.

**Figure 4:**
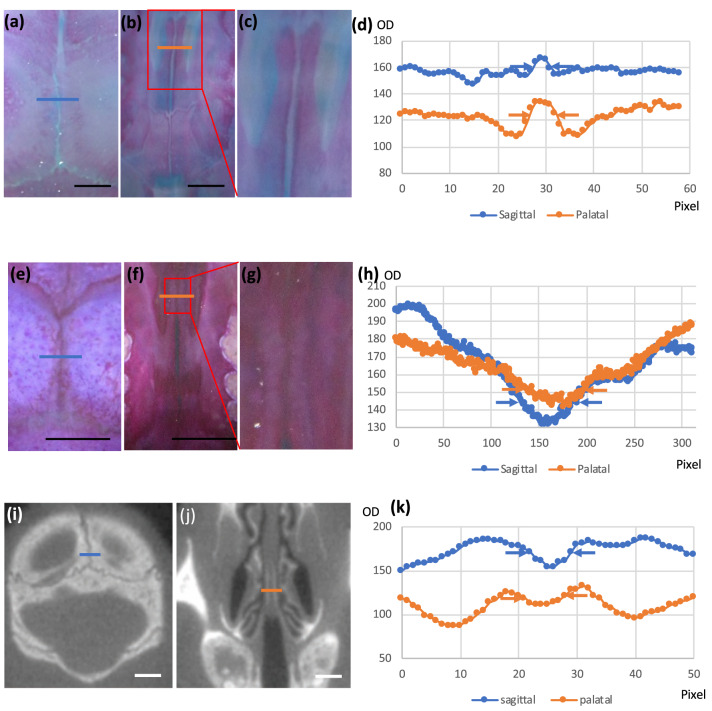
Sagittal and palatal suture widths are not significantly different in mice. **(a)** Sagittal suture of a juvenile mouse skull (P0). **(b)** Palatal suture of the same sample. **(c)** High-magnification view of **(b)**. **(d)** Profile plot of the suture line area. The difference between the sagittal suture and palatal suture of the juvenile mouse is not clear. **(e)** Sagittal suture of an adult mouse. **(f)** Palatal suture of the same adult mouse. **(g)** High-magnification view of **(f)**. **(h)** Profile plot of the suture line area. The difference between the sagittal and palatal suture of the adult mouse is not clear. **(i,j)** CTs of an adult mouse skull: **(i)** sagittal suture and **(j)** palatal suture. **(k)** CT values of the suture line area. The difference between the sagittal and palatal sutures of the mouse is not clear. Scale bars 1 mm.

We also compared the widths of the palatal and sagittal sutures of mice as a possible experimental animal. Juvenile ($n=3$) and adult ($n=3$) mice were sacrificed and stained with Alcian blue and Alizarin red , and the suture widths were compared by digitizing images of the sutures captured with a binocular microscope. There was no apparent difference between the widths of sagittal and palatal sutures in mice (Fig. 4a–h). To confirm the result, we also measured sagittal and palatal suture widths using 3DCT data obtained from the SIMBA Public Database provided by Cornell University ($n=3$). Again, we did not detect any difference between suture widths (Fig. 4i–k). Taken together, these findings suggest that width differences in sagittal and palatal sutures are species-dependent.

### Quantitative characteristics of palatal suture compared with sagittal suture

**Figure 5:**
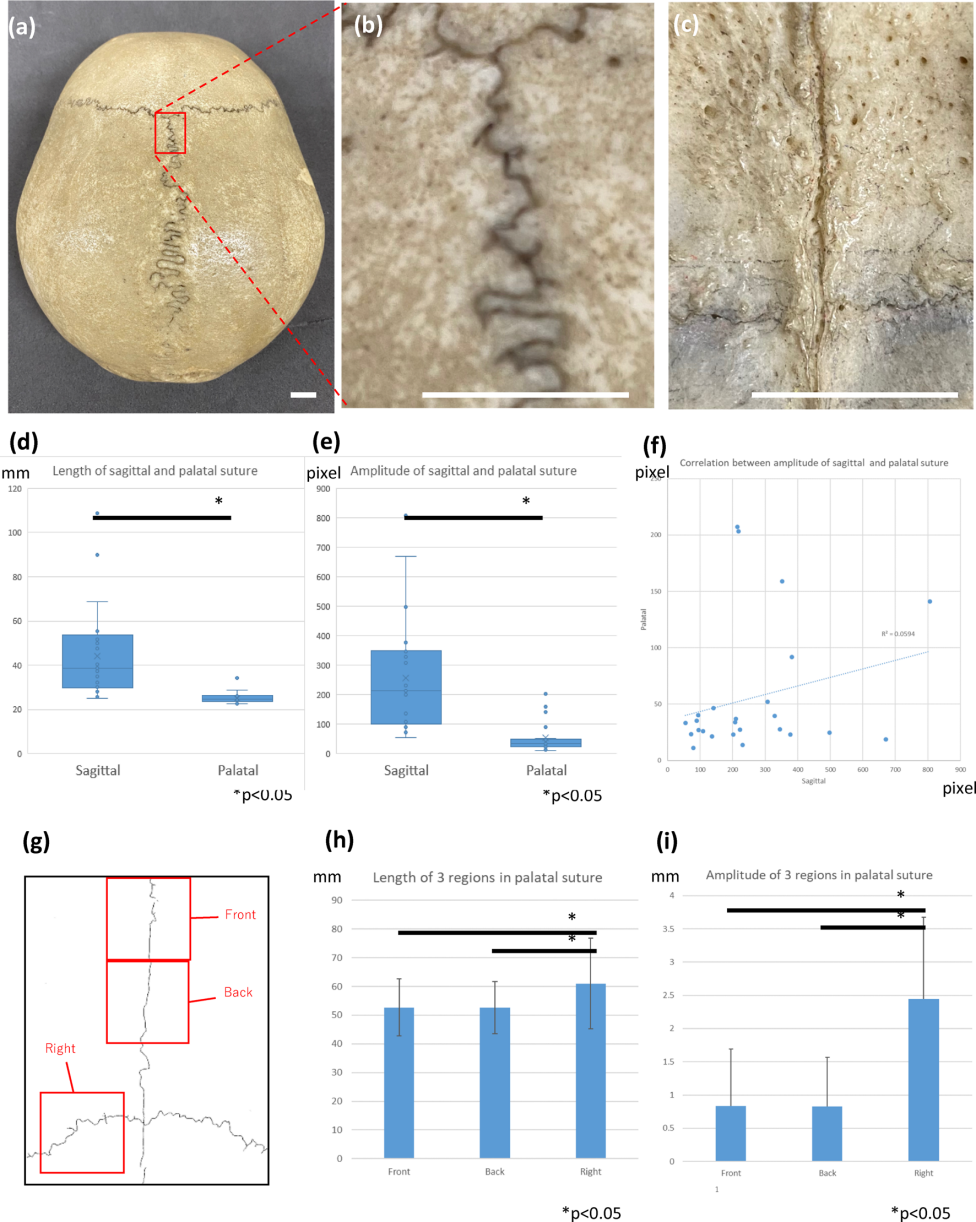
Measurement of differences depending on the site of palatal suture curvature. **(a)** Sagittal suture, low-magnification view. The curvature tends to be more pronounced at the rear along the longitudinal axis. **(b)** Sagittal suture, high-magnification view. **(c)** Palatal suture, high-magnification view. Curvature seems to be less pronounced. **(d)** Comparison of the lengths of sagittal and palatal sutures. The sagittal suture has a longer interdigitation length, indicating that winding is more prominent than in the palatal suture. **(e)** Comparison of the maximal width of the sagittal and palatal sutures. The sagittal suture has a wider interdigitation width. **(f)** Relationship between interdigitation values: a significant correlation did not exist between sagittal and palatal suture amplitudes ($R^2=0.0594$). **(g)** Regional differences in the palatal suture. **(h)** Comparison of the area of the skeletonized suture line. The lateral palatal suture was significantly longer than the other sutures. **(i)** Comparison of the maximum amplitude of palatal sutures. The curvature of the lateral palate suture showed significantly increased maximum width compared to that of the other sutures. Scale bars 10 mm.

We then quantified the characteristics of sagittal and palatal sutures using image analysis techniques ( $n=25$). First, we measured the length and amplitude of sagittal (Fig. 5a,b) and palatal sutures (Fig. 5c), both of which were larger in sagittal sutures (Fig. 5d,e). We also found that the correlation between sagittal and palatal suture amplitudes among individuals was not strong (Fig. 5f). For analysis of the local features of the palatal suture, we generated skeletonized images of three suture regions, in the front and back of the midline palatal suture and the right lateral palate suture, using ImageJ and then counted the number of pixels as a measure of suture length (Fig. 5). In addition, we determined the centerline of the suture position at the same site and considered the point farthest from this as the maximum amplitude: the larger the maximum amplitude, the larger the curvature of the suture (Fig. 5). Although we found no local difference in the midline suture, the lateral suture was longer and its curvature was larger than that of the midline suture (Fig. 5h). Furthermore, the amplitude of the transverse palatal suture was larger than that of the midline suture (Fig. 5i).

**Figure 6:**
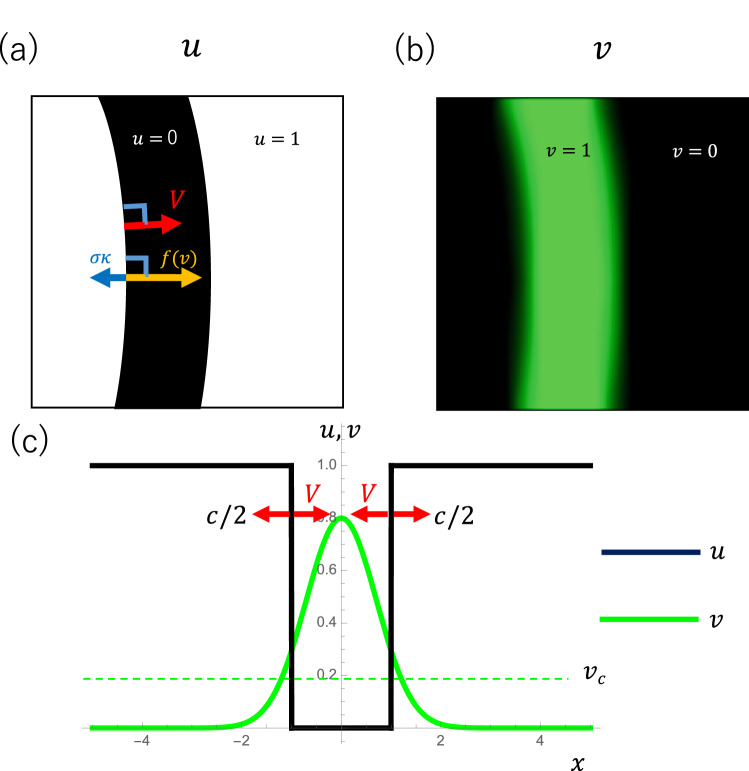
The theoretical model of suture patterning^31,32^. **(a)** Dynamics of bone differentiation degree *u*. The $u = 0$ section represents the soft tissue of the suture, whereas the $u = 1$ section represents the bone. The boundary between the bone and suture tissue moves at a speed *V* in the normal direction. The interface speed *V* is determined by the effect of differentiation factor *f*(*v*) and surface tension $\sigma \kappa$. **(b)** Distribution of bone differentiation promoting factor *v*: high in the suture tissue and low in the bone. **(c)** Schematic representation of the suture at a steady state. The osteogenic front speed *V* balances with the tissue expansion speed *c*/2. Therefore, *v* at the osteogenic front is slightly higher than $v_c$.

### Mathematical modeling of suture pattern formation

We used a mathematical model of suture development^31,32^ to further analyze suture pattern formation. In this model, we focused on the growth of the bone–mesenchyme interface and the signaling molecules that promote osteogenesis (i.e., FGF). We define *u*(*x*, *y*, *t*) as a bone shape (Fig. 6a) and *v* as a distribution of signaling molecules (Fig. 6b). We used the following assumptions in the model.

- Undifferentiated mesenchyme ($u=0$ region) produces osteopromoting diffusible signaling molecules *v*.
- Bone tends to differentiate with a high concentration of *v*. If the concentration exceeds a certain threshold of $v_c$, osteogenesis takes place. The efficacy of FGF on osteogenesis (the relationship between osteogenesis speed *V* and $v-v_c$) is separately defined as $\alpha$.
- Diffusible signaling molecule *v* is produced by mesenchyme cells ($u=0$ region), diffuses passively (diffusion coefficient $D_v$), and then decays.
- In the sagittal suture, the soft tissue region is passively expanded at speed *c*; as a result, soft mesenchyme tissue ($u=0$ region) grows horizontally.

We have previously reported that such interactions result in the spontaneous pattern formation of interdigitated suture structures^31,32^.

An intuitive explanation of the suture width maintenance and interdigitation formation is as follows: undifferentiated bone–mesenchyme produces diffusible osteopromoting factor *v*, which diffuses at diffusion coefficient $d_v$, and the osteogenic front moves according to the concentration of *v*. In such a system, the concentration of *v* at the interface determines the width of the suture. The efficacy $\alpha$ and critical concentration $v_c$ determine the speed of the osteogenic front. We define critical concentration $v_c$ as the concentration of *v* at which interface movement stops. These parameters determine and stably maintain the width of the suture. When the suture line is slightly curved, the protruded bone region should be exposed to a higher concentration of *v*, resulting in further protrusion formation. This effect amplifies initial slight perturbations of form and results in the formation of interdigitation. Surface tension $\sigma$ counteracts this effect. In addition, the suture is passively expanded by external factors such as brain growth, represented by *c*. In this case, suture width becomes a steady state when the interface speed *V* becomes balanced with the growth *c*/2 (Fig. 6c).

### Prediction of factors that influence suture width using mathematical modeling

**Figure 7:**
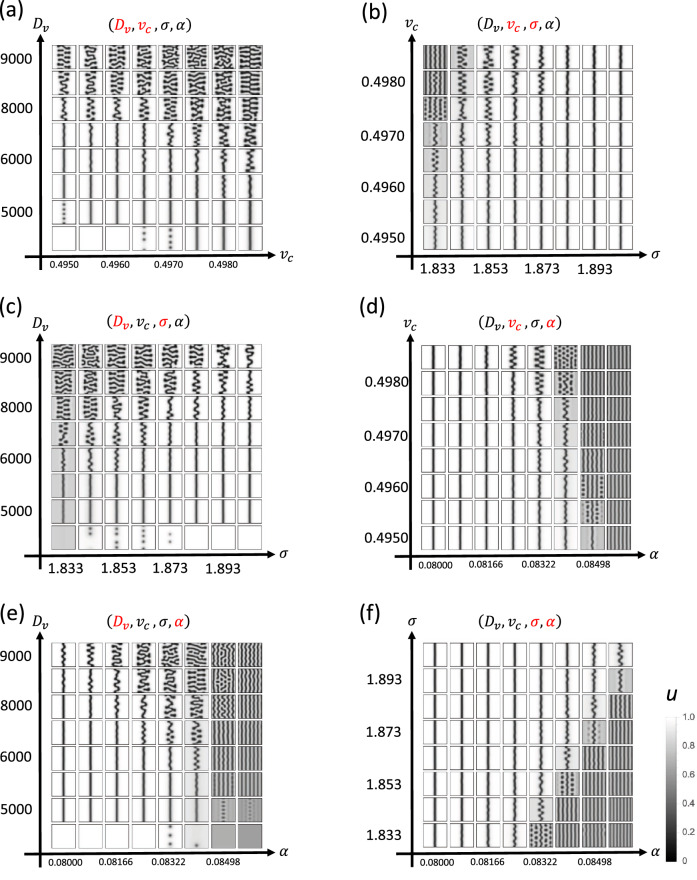
Numerical screening of possible factors that generate the differences between sagittal and palatal sutures. Four parameters ($D_v, v_c, \sigma , \alpha$) were selected and systematically changed to produce the phase diagrams.

**Figure 8:**
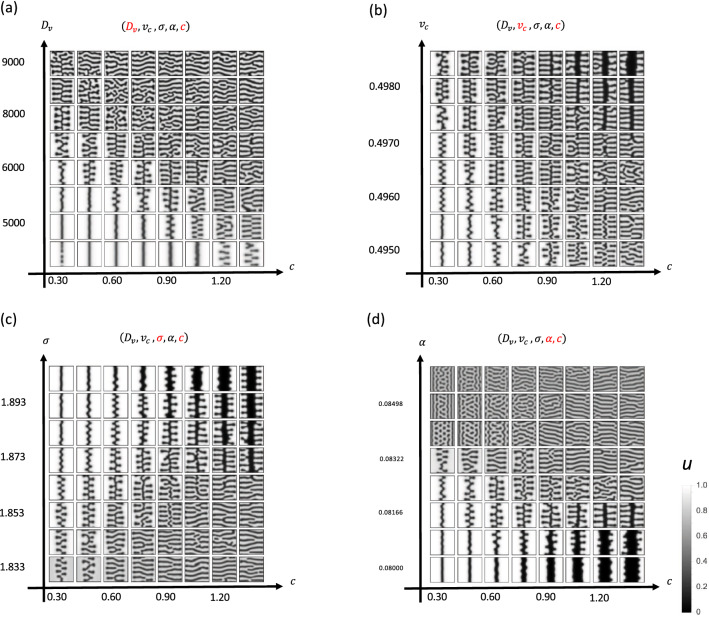
Numerical screening of possible factors that generate the differences between sagittal and palatal sutures. Four parameters ($D_v, v_c, \sigma , \alpha$) and growth speed *c* were selected and systematically changed to produce the phase diagrams.

We screened for the factors that produce the observed differences between palatal and sagittal sutures (Fig. 7, 8). According to our measurements, two major differences exist between sagittal and palatal sutures:

- Suture width
- Suture amplitude

In our model, we used five effective parameters:

- $\alpha$: Efficacy of FGF on osteogenesis
- $$\sigma $$: Surface tension
- $v_c$: Critical value of FGF (at this concentration, osteogenesis and bone resorption are balanced)
- $D_v$: Diffusion coefficient of FGF
- *c*: Growth speed of soft tissue

**Figure 9:**
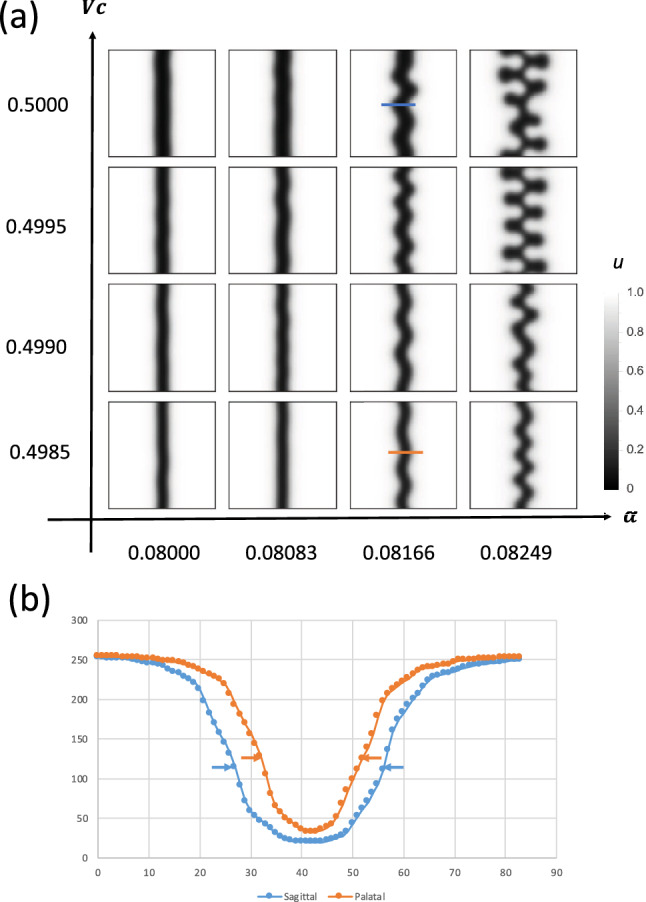
Change in critical FGF concentration ($v_c$) can account for sagittal and palatal suture differences. **(a)** Parameters were systematically changed to find parameter sets that could reproduce sagittal and palatal suture differences. **(b)** Suture width and interdigitation amplitude were increased by increasing $v_c$, which accounts for the difference between the suture types.

**Figure 10:**
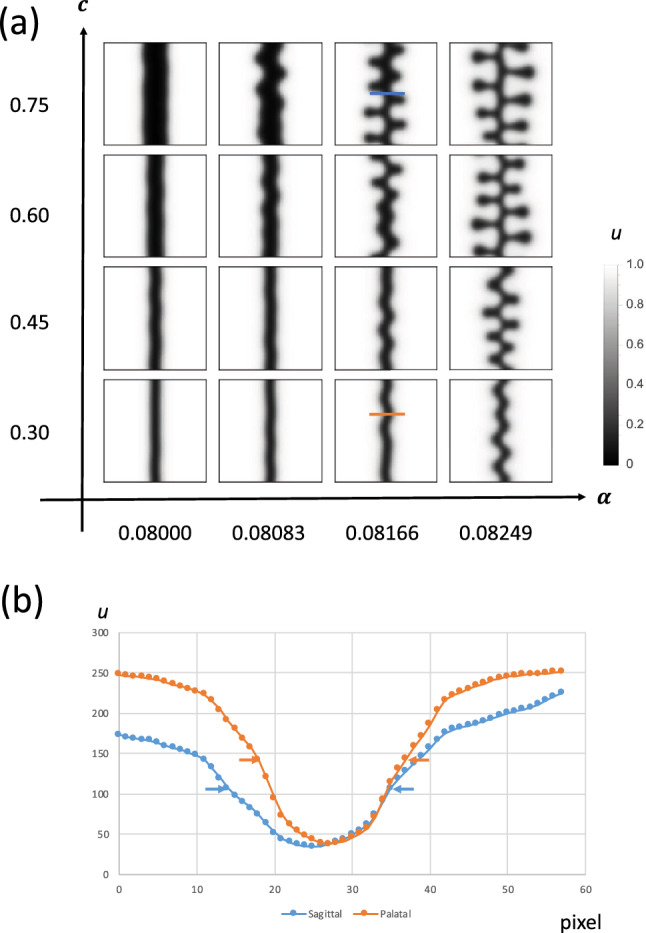
Passive expansion of parietal bones can result in a wider suture with increased amplitude. **(a)** Growth speed *c* and efficacy of FGF on osteogenesis $\alpha$ were systematically changed. **(b)** As the expansion speed *c* increased, the width and amplitude of interdigitation of the suture increased, which accounts for the difference between the palatal and sagittal suture.

Having screened this parameter set, we found that changing $v_c$ reproduced the two observable differences (Figs. 9, 10). Additionally, we examined whether the passive expansion of soft tissue could reproduce the observed changes. Accordingly, we implemented soft tissue growth by increasing the number of pixels at the minimum point of *v*. By doing so, we reproduced the wider suture width and larger amplitude of interdigitation, indicating that passive expansion of parietal bones is another cause of the morphological difference between sagittal and palatal sutures.

## Discussion

The results we obtained may be influenced by the variation of the samples due to the lack of detailed information on human specimens. The detailed information of the human skull collection (age, race, and gender) was not available. We chose samples in which sagittal sutures are patent. It is known that skull suture tends to fuse in older specimen^33^. Therefore the samples we used should represent the younger generation. Therefore we could not rule out the possibility that the older specimen may have a different tendency.

Our study may provide a cue to understand the difference between sagittal and palatal suture development. Dura mater lies beneath the sagittal suture, which may influence the suture patency. The previous organ culture study suggested that the dura mater secrete diffusive signaling molecules that maintain the suture tissue^34^. In our previous study, diffusible signaling molecule from the dura mater can enhance osteogenesis and inhibit suture interdigitation^32^. These two results seem to be inconsistent. However, we could not rule out that some parameter set satisfies both effects in the mathematical model. Further study is necessary to understand the effect of dura mater.

From our quantitative measurements, we did not detect local differences in the midline suture but found that the lateral suture was longer than the median palatal suture and that its curvature was larger than that of the midline suture. The length of the lateral suture may reflect the different types of transverse palatal suture previously reported^3^. The longer length of lateral suture may also be due to the mechanical effects of palatal palateus muscle, palatal levitation muscle, and vertical palateus muscle attachment to the palatal bone. Our model implements the effect of mechanical expansion by muscles, which can cause differences between sagittal and palatal sutures. Previous research has shown that the molecular response of sagittal and palatal sutures is similar^35^, and their response to mechanical stress is correlated to suture patency^21^. Mechanical stress is known to induce MMP-2 and TIMP during rapid maxillary expansion^36^. Indeed, the relationship between suture development and mechanics has recently been studied extensively^37,38^.

Species differences may shed light on the mechanism of palatal suture development. We observed no significant difference between the width of palatal and sagittal sutures in either chimpanzees or mice (Fig. 4). One of the factors that induced differences in our model was growth speed; the lack of suture differences in chimpanzee and mice skulls may, therefore, be due to the relatively slow growth of the calvaria. Comparing the characteristics of humans to other primates may provide further insights into the observed suture differences^39^. In addition, differences in maturation speed (sometimes known as heterochrony in evolutionary biology^39,40^) may also correlate with some of our model parameters and be useful for understanding species differences in suture development. Furthermore, ossification types may differ among species; for example, endochondral ossification occurs in the posterior part of the palate in mice^41^.

In our model, we identified two factors that could explain the difference between sagittal and palatal sutures. An intuitive explanation of how $v_c$ and *c* modify suture width and interdigitation amplitude is as follows: when $v_c$ is high, higher FGF concentration is necessary to promote osteogenesis; therefore, high $v_c$ reduces the osteogenic condition and results in a wider suture. Moreover, since competition for FGF at the bone interface generates interdigitation, a lower effective FGF concentration could increase the amplitude of suture interdigitation. A similar explanation could be applied to domain growth. When soft tissue is expanded at constant speed *c*, the suture width becomes stable when the bone interface speed is *c*/2. Consequently, undifferentiated suture tissue is wider and FGF concentration is higher than in cases with no growth.

Our mathematical model predicts that the main differences between sagittal and palatal suture are the critical values of FGF ($v_c$) and domain growth speed (*c*), both of which provide experimentally testable hypotheses. The value of $v_c$ should correspond to osteogenesis by a signaling molecule such as FGF. In humans, palatal sutures develop faster than other sutures^12^, which may reflect the observed differences. Concerning growth speed, the calvarial suture is passively expanded by the growth of the brain^11^; contrastingly, growth of the palatal suture is determined by the growth of the maxilla and palatal bone themselves. This difference may cause the palatal suture to be narrower relative to the sagittal suture.

The model we used is based on known molecular interactions of suture development^31,32,42^, but there are several other theoretical models of the pattern formation of cranial sutures. Some models are based on fractal geometry (Eden model^43^ and Diffusion-Limited Aggregation^43^). However, these models focused on the fractal nature of the pattern and did not implement the width of the suture lines, and are inappropriate to be used for the current work. Another class of models is based on mechanics^44^ instead of molecular pathways to implement interface instability. Mechanics and molecular biology are not mutually exclusive, and we do not deny that the mechanical aspect also plays a role.

In theoretical modeling of biological pattern formation, domain growth is of major interest as a Turing pattern modification factor^45^. Seminal work in fish skin patterns^46^ produced interest in domain growth, and various mathematical analyses have since been completed^47,48^. In our case, the growing region only occurred in soft tissue ($u=0$ region), which makes mathematical analysis difficult. However, since our governing equation is the interface equation, it should be possible to assess this domain growth mathematically. Moreover, our model may be able to explain the shape change induced by maxillary expansion. In a previous study, the expansion of the rat palatal suture resulted in the formation of a finger-like structure in the suture^22^; this may reflect the finger-like pattern we observed, as shown in Fig. 10.

## Methods

### Observation of human skull specimens

The adult human skull specimens used were bone specimens from a gross anatomy course at Kyushu University School of Medicine and Dentistry. These specimens were photographed at a fixed magnification and distance using a Nikon Coolpix P7000 connected to a stereomicroscope (Zeiss Stemi 2000CS). Pictures of newborn human skull specimens were provided by the laboratory of physical anthropology, Kyoto University. Juvenile chimpanzee skull specimens were provided by the Primate Research Institute, Kyoto University (KUPRI). This work was approved by Kyushu University Institutional Review Board for Clinical Research (2019-350).

### Observation of mouse skull specimens

Newborn (postnatal day 0: P0) and adult mice (ICR) were sacrificed by cervical dislocation and their skin was removed. The samples were then stained with Alcian blue and Alizarin red. Stained samples were cleared by immersion in glycerol and then images of the final samples were captured using a Nikon Coolpix P7000 connected to a stereomicroscope (Zeiss Stemi 2000CS). This experiment was undertaken with the permission of the Kyushu University animal experiment committee (A29-036-1). This experiment was carried out in compliance with the Kyushu University Animal Experiment Regulation and ARRIVE guidelines (http://www.nc3rs.org.uk/page.asp?id=1357).

We set the scale of the digitized images and undertook quantitative measurements using Fiji^49^.

### Observation of public domain CT data

Human CT volume data were obtained from Qure.ai (http://headctstudy.qure.ai/)^30^. Mouse microCT data were obtained from the SIMBA Public Database (http://www.via.cornell.edu/microdb.html ). For data visualization, OsiriX Lite (Pixmeo SARL Inc.) and SonicDICOM media viewer (JIUN Corporation) were used. 3DCT data of chimpanzee skulls were obtained from the Digital Morphology Museum, KUPRI (http://dmm.pri.kyoto-u.ac.jp/dmm/WebGallery/index.html).

### Numerical simulation of the model

Numerical simulation was performed using an implicit scheme. Lattice number: $128\times 128$, $\Delta x=50, \Delta t=2$. The simulation length was set to 6, 000. All numerical simulations were implemented in Python and NumPy on Google Colab (https://colab.research.google.com/). Source code is provided as supplementary electronic material.

## Supplementary Information

Supplementary Information.
